# Synthesis, crystal structure and Hirshfeld surface analysis of di­acetato­bis­[4-(2-amino­eth­yl)morpholine]cadmium tetra­hydrate

**DOI:** 10.1107/S2056989023008782

**Published:** 2023-10-19

**Authors:** B. Chidambaranathan, S. Sivaraj, P. Vijayamathubalan, S. Selvakumar

**Affiliations:** a PG and Research Department of Physics, Government Arts College for Men, (Autonomous), Chennai 600 035, Tamil Nadu, India; Universidad Nacional Autónoma de México, México

**Keywords:** crystal structure, coordination compound, morpholine ligand, crystal structure

## Abstract

The title coordination compound, [Cd(C_2_H_3_O_2_)_2_(C_6_H_14_N_2_O)_2_]·4H_2_O, was synthesized by mixing 2 moles of 4-(2-amino­eth­yl)morpholine and 1 mole of cadmium acetate in double-distilled water. The Cd atom is octa­hedrally coord­inated by two *N*,*N*′-bidentate ligands [4-(2-amino­eth­yl)morpholine] and two *trans*-located acetate mol­ecules. The Cd atom is located on a center of inversion, whereas the 4-(2-amino­eth­yl)morpholine and four water mol­ecules are adjacent to the acetate mol­ecules. The chair conformation of the morpholine mol­ecules is confirmed. In the crystal, adjacent metal complexes and uncoord­inated water mol­ecules are linked *via* N—H⋯O and O—H⋯O hydrogen-bonding inter­actions, generating *R*_2_^2^(6), *R*_6_^6^(16), *R*_6_^6^(20) and *S*_1_^1^(6) motifs and forming a three-dimensional network. A Hirshfeld surface analysis indicated the contributions of various contacts: H⋯H (71.8%), O⋯H/H⋯O (27.1%), and C⋯H/H⋯C (1.0%).

## Abstract 2

In the title coordination compound, the Cd atom is octa­hedrally coordinated by two *N*,*N*′-bidentate ligands [4-(2-amino­eth­yl)morpholine] and two *trans*-located acetate mol­ecules. The Cd atom is located on a centre of inversion, whereas the morpholine and four water mol­ecules are adjacent to the acetate moieties. In the crystal, neighboring metal complexes and uncoordinated water mol­ecules are linked *via* N—H⋯O and O—H⋯O hydrogen-bonding inter­actions.

## 1. Chemical context

Morpholine is generally recognized as a convenient ligand for the synthesis of a wide range of organometallic compounds (Beller *et al.*, 1999*a* ▸,*b* ▸) including discrete complexes (Stilinovic *et al.*, 2012 ▸) and metal–organic polymers (Sil Moon *et al.*, 2000 ▸). Although a morpholine mol­ecule is potentially an ambidentate N- and O- donor ligand, binding of morpholine to a metal centre is most commonly accomplished through the nitro­gen atom (Cvrtila *et al.*, 2012 ▸; Cindric *et al.*, 2013 ▸), except in cases where the nitro­gen atom is protonated (Li *et al.*, 2010 ▸; Willett *et al.*, 2005 ▸). Therefore, the oxygen atom can act as a halogen-bond acceptor (Lapadula *et al.*, 2010 ▸) or participate in hydrogen bonding (Weinberger *et al.*, 1998 ▸), among others, resulting in many different supra­molecular architectures. In the O⋯halogen bond, the O atom acts as an acceptor and the halogen (except F) acts as a donor.

The hydrogen atom of the secondary amino group can be easily substituted by an electrophilic species, allowing for the derivatization of morpholine to corresponding hydrazines (Johnson *et al.*, 2009 ▸), carbonyl compounds (Cheadle *et al.*, 2017 ▸; Tazi *et al.*, 2017 ▸) or Schiff bases (Hellmann *et al.*, 2019 ▸). A potentially inter­esting way of derivatizing the morpholine mol­ecule is carboxyl­ation of the nitro­gen atom, resulting in morpholine-*N*-carb­oxy­lic acid, or the respective morpholine-*N*-carboxyl­ate (Morph COO^−^) anion (Brown & Gray, 1981 ▸). This should act as an anionic ligand in coordinating metal ions through the carboxyl­ate group (Rao *et al.*, 2004 ▸). In a contin­uation of our recent work on compounds belonging to the morpholine family, we report here another compound in which morpholine is a ligand for a coordination complex. In the present study, a metal-coordinated compound of di­ace­t­ato­bis­[4-(2-amino­eth­yl)morpholine]­cadmium tetra­hydrate was synthesized and its structure was analysed by single crystal XRD. 
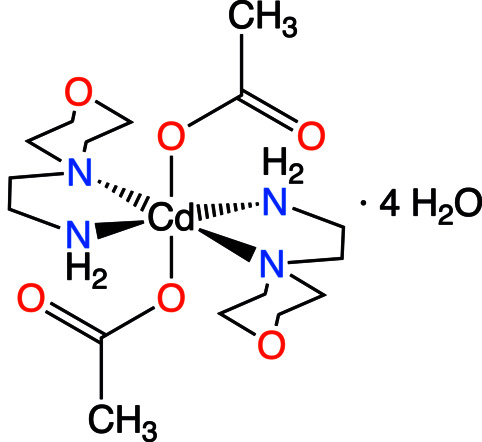


## 2. Structural commentary

**Figure 1:**
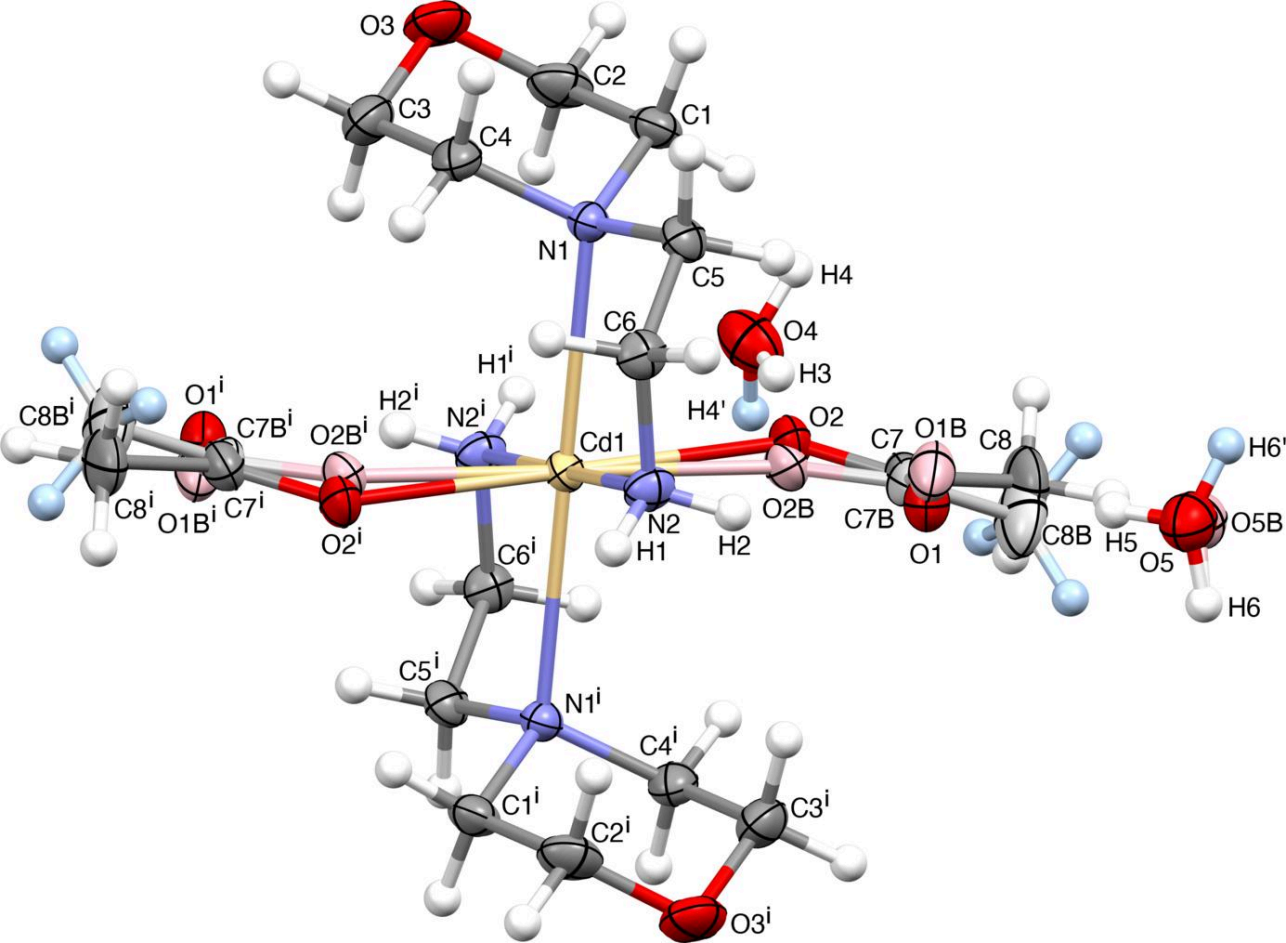
*ORTEP* diagram of the title compound with the atom-numbering scheme. Ellipsoids are drawn at 30% probability. [Symmetry code: (i) −*x* + 1, −*y*, −*z* + 1.]

**Figure 2:**
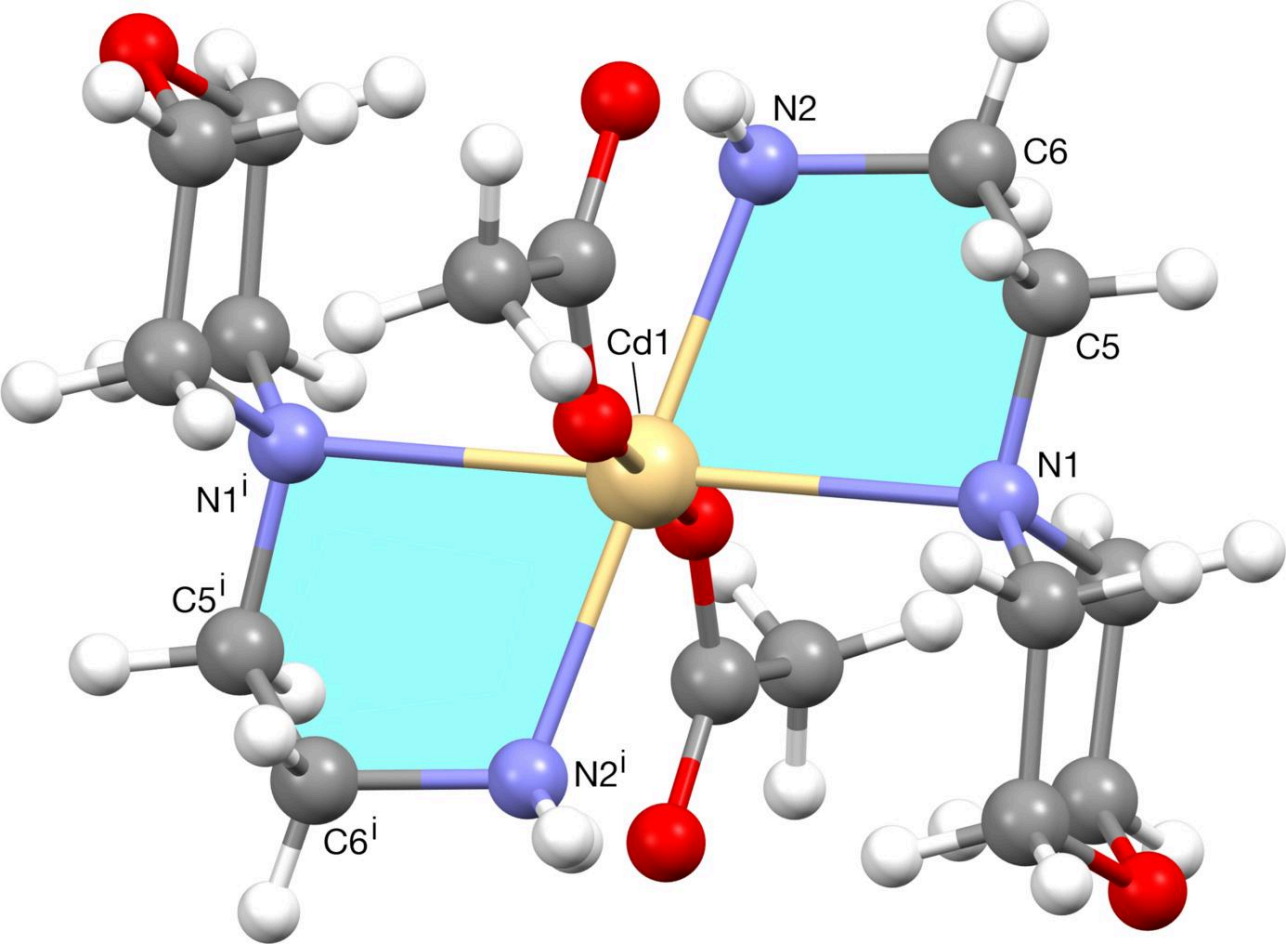
Five-membered chelate ring with metal as a centre. [Symmetry code: (i) −*x* + 1, −*y*, −*z* + 1.]

The title compound (Fig. 1 ▸) crystallizes in the triclinic crystal system, space group *P* 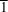 . The asymmetric unit comprises one-half of the Cd cation, which is located on an inversion centre, one [4-(2-amino­eth­yl)morpholine] ligand, one coordinated acetate anion and two water mol­ecules outside the metal coordination sphere. The structure consists of [Cd*L*_2_(OOCCH_3_)_2_]·4H_2_O units [where *L*= 4-(2-aminoeth­yl)morpholine]. The coordination polyhedron around the metal atom may be best described as a distorted octa­hedron. The four nitro­gen atoms of the di­amine ligands define the equatorial plane, and two oxygen atoms from the acetate anions coordinate in the *trans-*axial positions. The coordination of the morpholine ligands creates two five-membered chelate rings (Fig. 2 ▸). Upon coordination and formation of the five-membered chelate rings, these ligands are able to adapt themselves to the requirements of different metals (*M*) by varying the *M*—N distances and N—*M*—N angles. Many articles and reviews have reported that an important factor for metal-ion selection is the chelate ring size, in which five-membered chelate rings promote selectivity for large metal ions with an ionic radius (*r* ^+^) close to 1.0 Å. Theoretical calculations show that for five-membered N–C–C–N–*M* chelate rings, the ideal values for the N—*M* distance and N—*M*—N angle are 2.5 Å and 69°, respectively (Hancock 1992 ▸; Hancock *et al.*, 2007 ▸; Dean *et al.*, 2008 ▸). An inverse relationship exists between the *M*—N bond length and the N—*M*—N bond angle in the five-membered chelate rings, meaning that the variation of the N—*M*—N angle is directly related to the *M*—N bond length (Baza­rgan *et al.*, 2019 ▸). In the present study, the Cd—N (amine) distances are 2.5239 (13) Å (Cd—N1 and Cd—N1^i^) and 2.2788 (15) Å (Cd—N2 and Cd—N2^i^), are in good agreement with the values reported in the literature (Chiumia *et al.*, 1999 ▸; Chattopadhyay *et al.*, 2005 ▸). The substantial difference in their values is a consequence of the steric constraints imposed by the bulky morpholine group. As a result of symmetry, the N2—Cd1—N2^i^, N1—Cd1—N1^i^ and O2—Cd1—O2^i^ angles are 180° [symmetry code: (i) −*x* + 1, −*y*, −*z* + 1] and the *cis*-angles of the octa­hedron involving O2 and O2^i^ are close to the ideal value of 90°. The morpholine rings adopt a chair conformation. The acetate group is disordered over two positions of equal occupancy and in both of the crystallographically independent water mol­ecules, one of the protons is equally disordered over two positions. Finally, water atom O5 from the water mol­ecules is disordered over two positions in a 75 (3):25 (3) ratio.

## 3. Supra­molecular features

**Figure 3:**
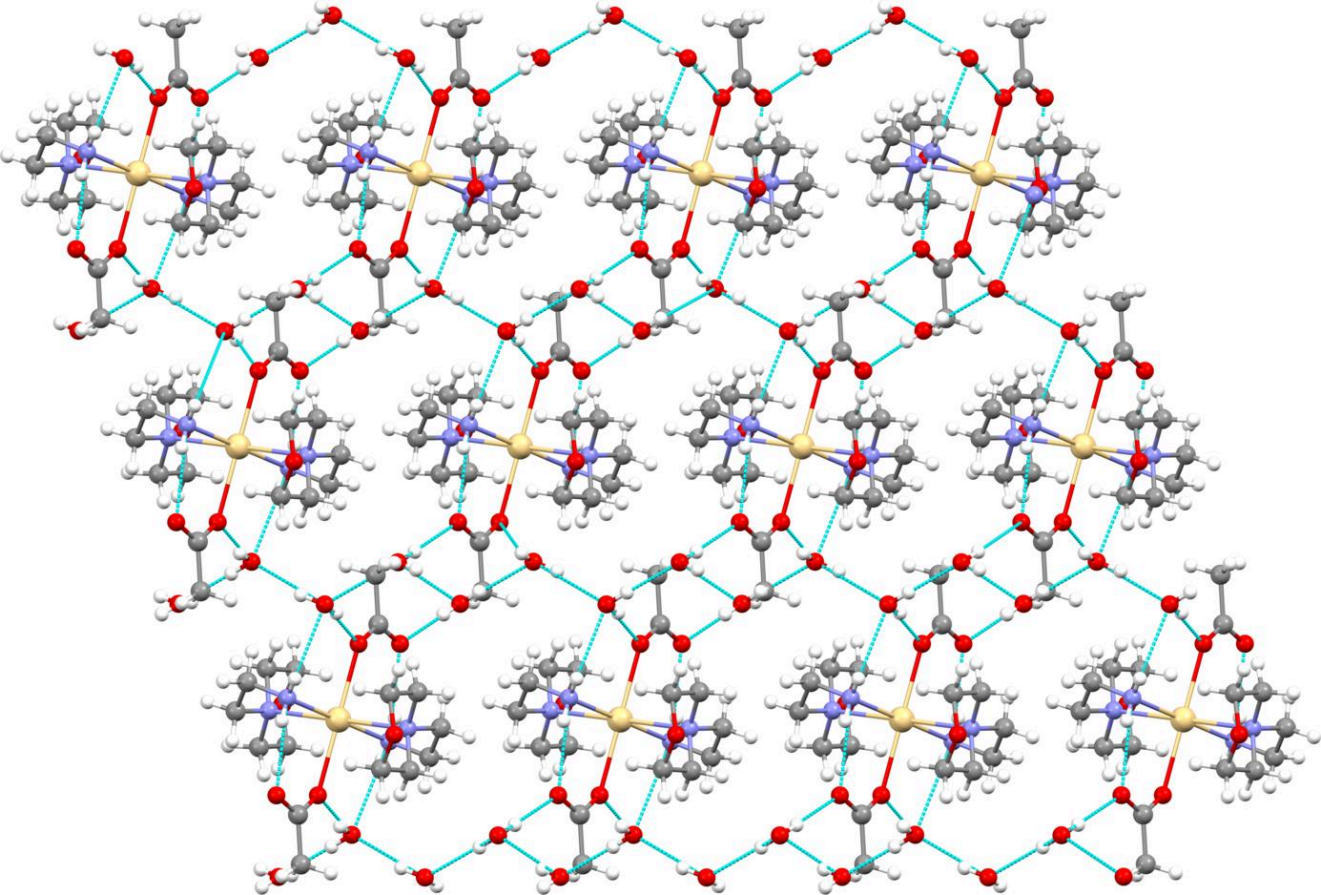
Crystal packing diagram of the title compound along the *a* axis.

**Figure 4:**
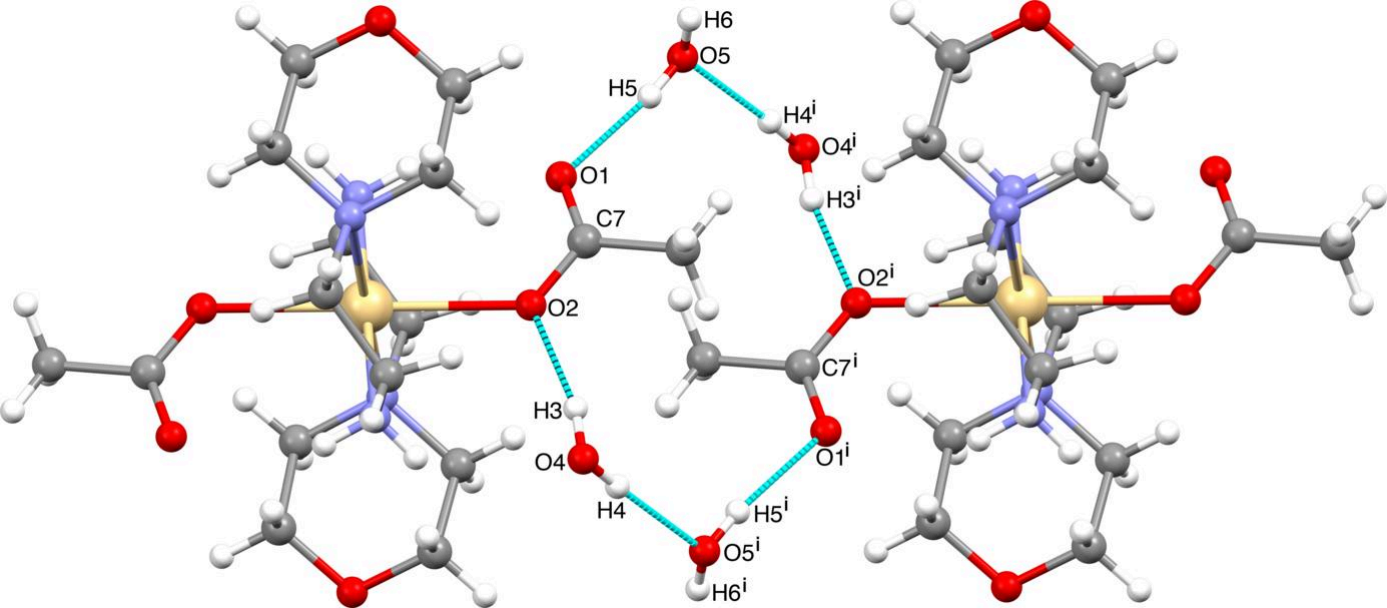
Inter­molecular inter­actions forming the 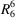 (16) ring motif. [Symmetry code: (i) 1 − *x*, 1 − *y*, −*z*.]

**Figure 5:**
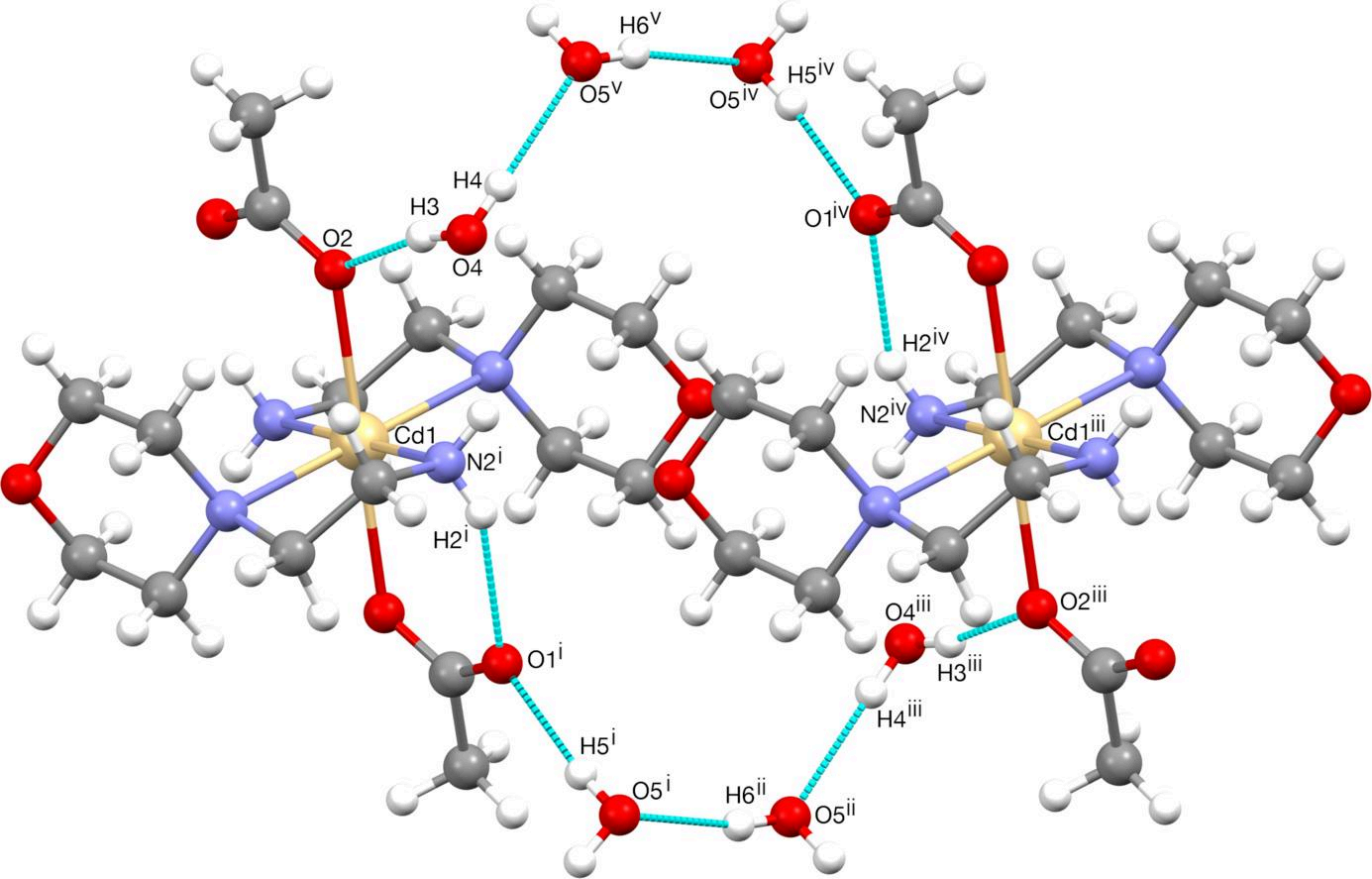
Inter­molecular inter­actions forming the 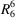 (20) ring motif. [Symmetry codes: (i) −*x* + 1, −*y*, −*z* + 1, (ii) −*x* + 1, −*y* + 1, *z* + 1, (iii) −*x*, −*y*, −*z* + 1, (iv) *x* − 1, *y*, *z*, (v) 1 − *x*, 1 − *y*, −*z*.]

**Figure 6:**
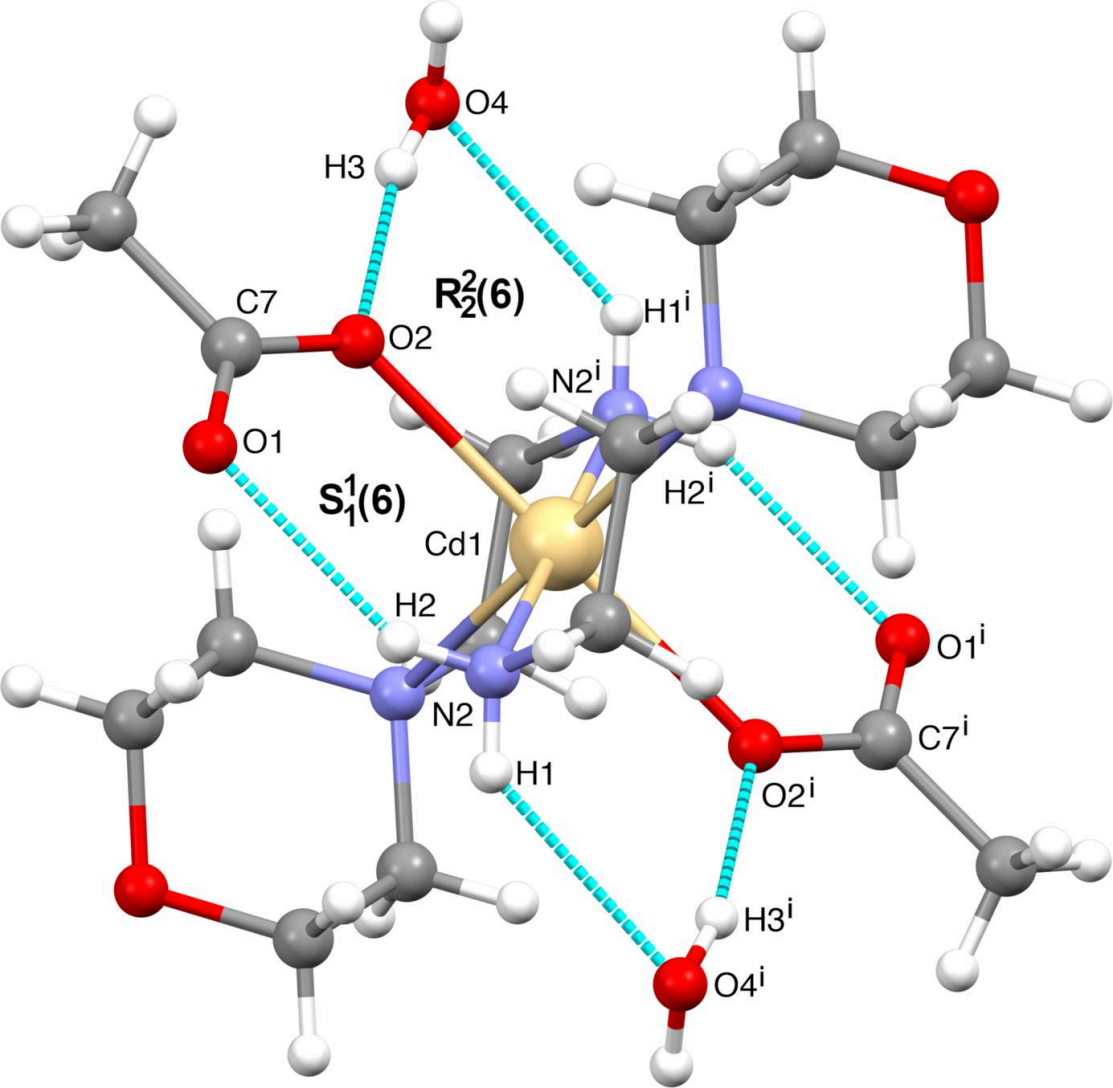
The N—H⋯O intra­molecular inter­action forming an 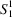 (6) motif.

**Figure 7:**
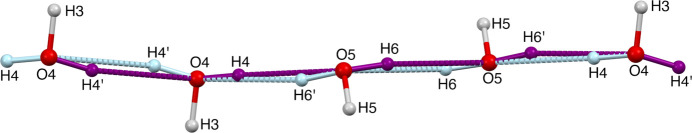
Two different orientations of the hydrogen bonds connecting the water mol­ecules into infinite chains running in opposite directions.

Hydrogen bonding is the most dominant mechanism for mol­ecular recognition. Graph-set analysis potentially provides the tools for a systematic analysis of the patterns of hydrogen-bonded networks. Hydrogen-bond pattern functionality might then be employed to predict the three-dimensional structure of a compound or to design substances with a desired and predetermined structure (Bernstein *et al.*, 1995 ▸; Motherwell *et al.*, 2000 ▸). The crystal packing of the title compound is shown in Fig. 3 ▸, illustrating the infinite chain structure formed through a hydrogen-bonding network along the *a*-axis direction indicated by cyan dashed lines. In the crystal, the mol­ecules are linked by numerous N—H⋯O and O—H⋯O inter­actions (Table 1 ▸), enclosing 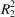 (6), 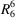 (16) and 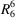 (20) ring motifs. Fig. 4 ▸ shows the 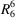 (16) ring motif formed by O4—H3⋯O2, O4—H4⋯O5 and O5—H5⋯O1 hydrogen bonds while the N2—H2⋯O1, O5—H5⋯O1, O4—H4⋯O5 and O4—H3⋯O2 inter­actions form an 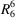 (20) ring motif (Fig. 5 ▸). Fig. 6 ▸ illustrates the 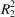 (6) ring formed between the complex and the O4-containing water mol­ecule *via* O4—H3⋯O2 and N2—H1⋯O4^i^ hydroge bonds. Finally, the mol­ecular structure is stabilized by an intra­molecular N2—H2⋯O1 hydrogen bond, which forms an 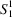 (6) motif (Fig. 6 ▸). These inter­actions link the mol­ecules into a three-dimensional network. For the sake of clarity, the figures show only one position of the disordered moieties. While the disorder of the acetate group or O5 does not change significantly the hydrogen-bond pattern, the disorder of the water protons H4 and H6 creates two different orientations of the hydrogen bonds connecting the water mol­ecules into infinite chains running in opposite directions, as depicted in Fig. 7 ▸.

**Figure 8:**
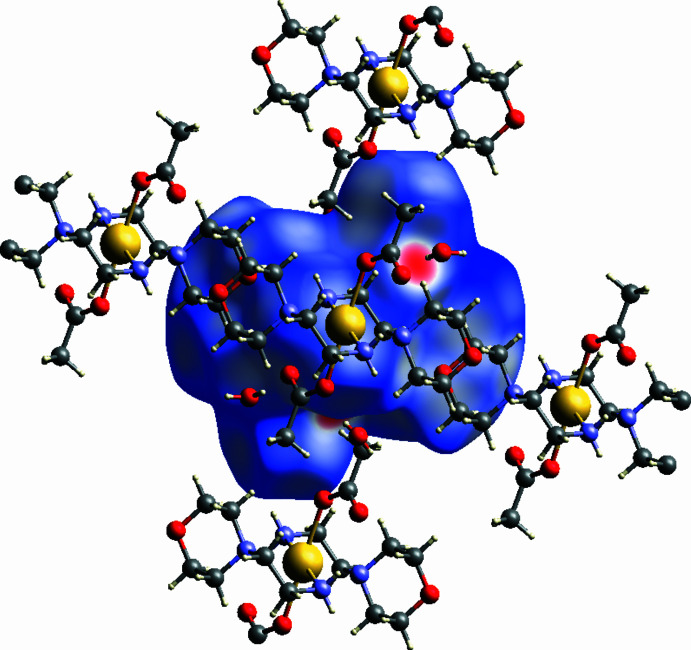
The Hirshfeld surface of the title compound mapped over *d*_norm_, showing the relevant close contacts.

**Figure 9:**
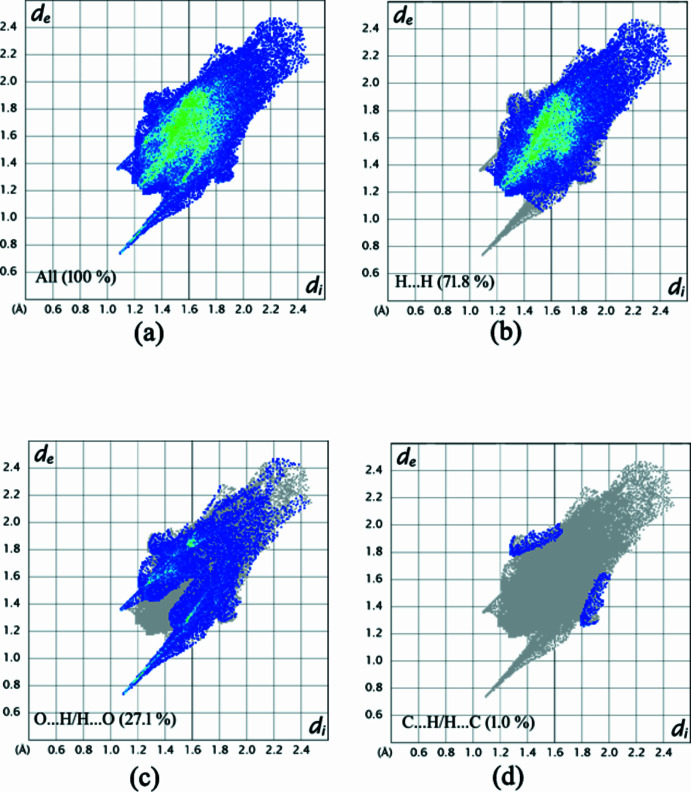
Two-dimensional fingerprint plots for the title compound, showing (*a*) all inter­actions, and delineated into (*b*) H⋯H, (*c*) O⋯H/H⋯O and (*d*) C⋯H/H⋯C inter­actions.

A Hirshfeld surface analysis was performed for the complex alone (excluding the water molecules) and the two-dimensional (2D) fingerprint plots were created with *Crystal Explorer 21.5* (Spackman *et al.*, 2021 ▸; McKinnon *et al.*, 2007 ▸). The Hirshfeld surface mapped over *d*_norm_, in the range −0.5934 to 1.4137 a.u is shown in Fig. 8 ▸ where red spots on the Hirshfeld surface indicate hydrogen bonds. The two-dimensional fingerprint plots illustrate the distribution of the different inter­actions (Fig. 9 ▸). H⋯H inter­actions (Fig. 9 ▸ *b*) are the most significant, contributing 71.8% to the total crystal packing. This major contribution may be due to van der Waals inter­actions (Hathwar *et al.*, 2015 ▸). The next most frequent inter­action is O⋯H/H⋯O (27.1%) (Fig. 9 ▸ *c*). Fig. 9 ▸ *d* shows the C⋯H/H⋯C inter­actions, which contribute 1.0% to the Hirshfeld surface.

## 4. Database survey

A search in the Cambridge Structural Database (CSD, version 5.40; Groom *et al.*, 2016 ▸) for 4-(2-amino­eth­yl)morpholine yielded eleven hits for coordination compounds of 4-(2-amino­eth­yl)morpholine with metals, including *catena*-[bis­(μ_2_-dicyanamide-*N*,*N*′)-[4-(2-amino­eth­yl)morpholine]]­nickel(II) (FIJROG; Konar *et al.*, 2005 ▸), bis­[2-(morpholin-4-yl)ethan­amine)(5,10,15,20-tetra­kis­(4-meth­oxy­phen­yl)porphyrinato]iron(II) (NABXEW; Ben Haj Hassen *et al.*, 2016 ▸; NABXEW01; Khelifa *et al.*, 2016 ▸), *trans*-bis­[4-(2-amino­eth­yl)morpholine]­bis­(nitrito)nickel(II) (NAVNAA; Chattopadhyay *et al.*, 2005 ▸; RANVEJ and NAVNAA01; Brayshaw *et al.*,2012 ▸), *trans*-bis­(iso­thio­cyanato-*N*)bis­[4-(2-amino­eth­yl)morpholine-*N*,*N*′]nickel(II) (NENSUU; Laskar *et al.*, 2001 ▸), 4-[(2-amino­eth­yl)morpholine-*N*,*N*′]aqua­(oxalato-*O*,*O*′)copper(II) monohydrate (XAZRUM; Koćwin-Giełzak & Marciniak *et al.*, 2006 ▸), (μ_2_-oxalato)bis­[4-(2-amino­eth­yl)morpholine]­di­cop­per(II) (YIKQAK; Mukherjee *et al.*, 2001 ▸), di­chloro-bis­(2-morpholine-4-yl)ethanamine­cadmium(II) (ULAJEX; Sulei­man Gwaram *et al.*, 2011 ▸) and *trans*-di­aqua­bis­[4-(2-amino­eth­yl)morpholine-*κ^2^*-*N*,*N*′]nickel(II) dichloride (VEPHIL; Chidambaranathan *et al.*, 2023 ▸). It is found that all of these structures are stabilized by hydrogen bonds. The morpholine ring adopts a chair conformation, and the amine functions as an *N*,*N*′-bidentate ligand to form a five-membered chelate ring with the metal centre, as observed with the other metal complexes of 4-(2-amino­eth­yl)morpholine.

## 5. Synthesis and crystallization

**Figure 10:**
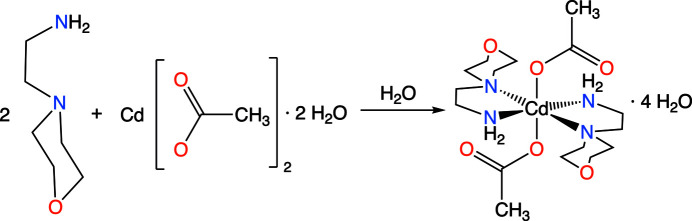
Synthesis of the title compound.

As shown in the reaction scheme (Fig. 10 ▸), the title compound was synthesized by mixing two moles of 4-(2-amino­eth­yl)morpholine (2.40 g) and one mole of cadmium acetate (2.67 g) in 150 ml of double-distilled water at 303 K. The solution was allowed to evaporate at room temperature and needle-like crystals of the title compound were obtained. The FT–IR spectrum of the compound was recorded on a Bruker FT–IR spectrometer. FT–IR (KBr, cm^−1^): 3301 (*w*, OH), 2887 (*w*, CH_2_), 1549 (*s*, NH), 1411 (*s*, C—C), 1342 (*s*, C—N), 1192 (*w*, C—N), 960 (*w*, C—O), 653 (*s*, OH_2_) and 594 (*s*, *M*—N).

## 6. Refinement

Crystal data, data collection and structure refinement details are summarized in Table 2 ▸. All C—H atoms were positioned geometrically (C—H = 0.96–0.97 Å) and refined as riding with *U*_iso_(H) = 1.2–1.5*U*_eq_(C), while the N—H and O—H protons were located in residual electron-density maps and refined with distance restraints (DFIX and SADI) and with *U*_iso_(H) = 1.2*U*_eq_(N) and 1.5*U*_eq_(O). The acetate group was refined as disordered over two positions (ratio 50:50%) with distance, geometry and *U_ij_* restraints (SADI, FLAT, SIMU and RIGU). H4 and H6 are disordered over two positions in a 50:50 ratio due to symmetry-related hydrogen bonds. O5 is disordered over two positions in a 75 (3):25 (3) ratio. As both positions have the same distance to H5, H6 and H6′, only one set of the hydrogen atoms was refined for both O5 and O5*B*.

## Supplementary Material

Crystal structure: contains datablock(s) I. DOI: 10.1107/S2056989023008782/jq2030sup1.cif

Structure factors: contains datablock(s) I. DOI: 10.1107/S2056989023008782/jq2030Isup4.hkl

CCDC reference: 2299387

Additional supporting information:  crystallographic information; 3D view; checkCIF report

## Figures and Tables

**Table 1:** Hydrogen-bond geometry (Å, °)

*D*—H⋯*A*	*D*—H	H⋯*A*	*D*⋯*A*	*D*—H⋯*A*
C4—H4*B*⋯O2*B* ^i^	0.97	2.66	3.264 (19)	121
C5—H5*B*⋯O1*B*	0.97	2.63	3.425 (16)	139
N2—H1⋯O4^i^	0.84 (2)	2.44 (2)	3.096 (3)	136 (2)
N2—H2⋯O1	0.84 (2)	2.26 (2)	3.009 (16)	147 (2)
N2—H2⋯O1*B*	0.84 (2)	2.26 (3)	2.993 (17)	145 (2)
O4—H3⋯O2	0.83 (2)	1.84 (3)	2.661 (17)	167 (4)
O4—H3⋯O2*B*	0.83 (2)	2.02 (3)	2.837 (17)	165 (3)
O4—H4⋯O5^ii^	0.82 (5)	2.06 (5)	2.879 (7)	178 (7)
O4—H4⋯O5*B* ^ii^	0.82 (5)	2.05 (5)	2.847 (18)	165 (5)
O4—H4′⋯O4^iii^	0.83 (2)	2.11 (3)	2.917 (4)	164 (6)
O5—H5⋯O1	0.84 (2)	1.99 (2)	2.828 (16)	173 (3)
O5*B*—H5⋯O1*B*	0.83 (2)	1.93 (2)	2.70 (2)	155 (3)
O5—H6⋯O5^iv^	0.82 (2)	2.09 (3)	2.895 (13)	166 (5)
O5—H6⋯O5*B* ^iv^	0.82 (4)	1.93 (5)	2.75 (2)	173 (5)
O5—H6′⋯O4^ii^	0.83 (2)	2.07 (2)	2.878 (5)	168 (5)
O5*B*—H6′⋯O4^ii^	0.84 (2)	2.07 (2)	2.846 (17)	154 (5)

Symmetry codes: (i) 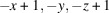 ; (ii) 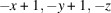 ; (iii) 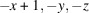 ; (iv) 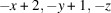 .

**Table 2:** Experimental details

Crystal data	
Chemical formula	[Cd(C_2_H_3_O_2_)_2_(C_6_H_14_N_2_O)_2_]·4H_2_O
*M*_r_	562.93
Crystal system, space group	Triclinic, *P* 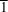
Temperature (K)	296
*a*, *b*, *c* (Å)	8.8639 (4), 9.1035 (5), 9.2106 (5)
α, β, γ (°)	66.004 (2), 73.603 (2), 70.161 (2)
*V* (Å^3^)	629.63 (6)
*Z*	1
Radiation type	Mo *K*α
μ (mm^−1^)	0.92
Crystal size (mm)	0.42 × 0.25 × 0.20

Data collection	
Diffractometer	Bruker APEXII
Absorption correction	Multi-scan (*SADABS*; Krause *et al.*, 2015 ▸)
*T*_min_, *T*_max_	0.603, 0.746
No. of measured, independent and observed [*I* > 2σ(*I*)] reflections	19482, 3034, 3024
*R*_int_	0.065
(sin θ/λ)_max_ (Å^−1^)	0.660

Refinement	
*R*[*F* ^2^ > 2σ(*F* ^2^)], *wR*(*F* ^2^), *S*	0.022, 0.056, 1.03
No. of reflections	3034
No. of parameters	209
No. of restraints	156
H-atom treatment	H atoms treated by a mixture of independent and constrained refinement
Δρ_max_, Δρ_min_ (e Å^−3^)	0.35, −0.41

Computer programs: *APEX2*, *SAINT* and *XPREP* (Bruker, 2004 ▸), *SHELXT2018/2* (Sheldrick, 2015*a* ▸), *SHELXL2019/2* (Sheldrick, 2015*b* ▸) and *Mercury* (Macrae *et al.*, 2020 ▸).

## supplementary crystallographic information

### Crystal data

**Table:**

[Cd(C_2_H_3_O_2_)_2_(C_6_H_14_N_2_O)_2_]·4H_2_O	*Z* = 1
*M**_r_* = 562.93	*F*(000) = 294
Triclinic, *P*1	*D*_x_ = 1.485 Mg m^−^^3^
*a* = 8.8639 (4) Å	Mo *K*α radiation, λ = 0.71073 Å
*b* = 9.1035 (5) Å	Cell parameters from 8460 reflections
*c* = 9.2106 (5) Å	θ = 2.5–27.7°
α = 66.004 (2)°	µ = 0.92 mm^−^^1^
β = 73.603 (2)°	*T* = 296 K
γ = 70.161 (2)°	BLOCK, yellow
*V* = 629.63 (6) Å^3^	0.42 × 0.25 × 0.20 mm

### Data collection

**Table:**

Bruker APEXII diffractometer	3034 independent reflections
Radiation source: fine-focus sealed tube	3024 reflections with *I* > 2σ(*I*)
Graphite monochromator	*R*_int_ = 0.065
Detector resolution: 8.333 pixels mm^-1^	θ_max_ = 28.0°, θ_min_ = 2.5°
ω and φ scan	*h* = −11→11
Absorption correction: multi-scan (SADABS; Krause *et al.*, 2015)	*k* = −12→12
*T*_min_ = 0.603, *T*_max_ = 0.746	*l* = −12→12
19482 measured reflections	

### Refinement

**Table:**

Refinement on *F*^2^	Secondary atom site location: difference Fourier map
Least-squares matrix: full	Hydrogen site location: mixed
*R*[*F*^2^ > 2σ(*F*^2^)] = 0.022	H atoms treated by a mixture of independent and constrained refinement
*wR*(*F*^2^) = 0.056	*w* = 1/[σ^2^(*F*_o_^2^) + (0.0252*P*)^2^ + 0.0883*P*] where *P* = (*F*_o_^2^ + 2*F*_c_^2^)/3
*S* = 1.03	(Δ/σ)_max_ < 0.001
3034 reflections	Δρ_max_ = 0.35 e Å^−^^3^
209 parameters	Δρ_min_ = −0.41 e Å^−^^3^
156 restraints	Extinction correction: *SHELXL2019/2* (Sheldrick 2015b), Fc^*^=kFc[1+0.001xFc^2^λ^3^/sin(2θ)]^-1/4^
Primary atom site location: structure-invariant direct methods	Extinction coefficient: 0.070 (3)

### Special details

**Table:**

Geometry. All esds (except the esd in the dihedral angle between two l.s. planes) are estimated using the full covariance matrix. The cell esds are taken into account individually in the estimation of esds in distances, angles and torsion angles; correlations between esds in cell parameters are only used when they are defined by crystal symmetry. An approximate (isotropic) treatment of cell esds is used for estimating esds involving l.s. planes.
Refinement. Acetate moiety is disordered over two positions with an occupancy ratio of 1:1. SADI restraint was used to fix similar distances to be equal for the disordered atoms. The anisotropic displacement parameters of atoms in the disordered groups were restrained to be equal with an effective standard deviation of 0.02A2 using SIMU restraint. Hydrogen, H6, on O5 water molecules is having two possible locations labelled as H6 and H6'. The refined occupancy ratio of H6 and H6' was found to be 47:53

### Fractional atomic coordinates and isotropic or equivalent isotropic displacement parameters (Å^2^)

**Table:**

	*x*	*y*	*z*	*U*_iso_*/*U*_eq_	Occ. (<1)
Cd1	0.500000	0.000000	0.500000	0.02834 (8)	
C1	0.1597 (2)	0.3173 (2)	0.3992 (2)	0.0414 (4)	
H1A	0.234102	0.335992	0.297375	0.050*	
H1B	0.092779	0.424930	0.404315	0.050*	
C2	0.0527 (3)	0.2171 (3)	0.4051 (3)	0.0526 (5)	
H2A	−0.011008	0.279160	0.318855	0.063*	
H2B	0.120004	0.114484	0.388209	0.063*	
C3	0.0405 (3)	0.0877 (3)	0.6807 (3)	0.0525 (5)	
H3A	0.110930	−0.013930	0.663716	0.063*	
H3B	−0.031524	0.057256	0.783056	0.063*	
C4	0.1428 (2)	0.1877 (2)	0.6869 (2)	0.0410 (4)	
H4A	0.072480	0.287436	0.707912	0.049*	
H4B	0.204906	0.122555	0.774156	0.049*	
C5	0.3357 (2)	0.3536 (2)	0.5304 (2)	0.0378 (4)	
H5A	0.254126	0.441193	0.564346	0.045*	
H5B	0.386251	0.404292	0.420633	0.045*	
C6	0.4629 (2)	0.2741 (2)	0.6376 (2)	0.0371 (4)	
H6A	0.504495	0.359169	0.639032	0.044*	
H6B	0.414465	0.218855	0.746809	0.044*	
N1	0.25477 (17)	0.23413 (17)	0.53308 (17)	0.0309 (3)	
N2	0.59686 (19)	0.1529 (2)	0.5791 (2)	0.0373 (3)	
H1	0.657 (3)	0.101 (3)	0.649 (3)	0.045*	
H2	0.654 (3)	0.202 (3)	0.495 (2)	0.045*	
C7	0.6344 (19)	0.2664 (19)	0.1517 (17)	0.046 (2)	0.5
C8	0.667 (2)	0.321 (2)	−0.0301 (15)	0.072 (3)	0.5
H8A	0.565533	0.368190	−0.069697	0.108*	0.5
H8B	0.727822	0.226857	−0.062787	0.108*	0.5
H8C	0.728342	0.402997	−0.073442	0.108*	0.5
O1	0.6956 (18)	0.3179 (17)	0.2228 (18)	0.0498 (19)	0.5
O2	0.539 (2)	0.171 (2)	0.223 (2)	0.045 (3)	0.5
C7B	0.6416 (18)	0.2682 (19)	0.1635 (16)	0.046 (2)	0.5
C8B	0.707 (2)	0.297 (2)	−0.0148 (15)	0.079 (4)	0.5
H8B1	0.647320	0.403671	−0.077770	0.119*	0.5
H8B2	0.694369	0.211533	−0.042399	0.119*	0.5
H8B3	0.819967	0.295000	−0.036825	0.119*	0.5
O1B	0.6664 (19)	0.3510 (17)	0.228 (2)	0.055 (3)	0.5
O2B	0.566 (2)	0.154 (2)	0.233 (2)	0.0407 (19)	0.5
O3	−0.05347 (18)	0.1793 (2)	0.5549 (2)	0.0623 (4)	
O4	0.3639 (3)	0.1019 (2)	0.0781 (2)	0.0653 (5)	
H3	0.424 (4)	0.133 (4)	0.109 (4)	0.098*	
H4	0.302 (6)	0.190 (4)	0.034 (8)	0.098*	0.5
H4'	0.431 (5)	0.030 (7)	0.044 (8)	0.098*	0.5
O5	0.8474 (6)	0.5833 (6)	0.0761 (12)	0.0584 (13)	0.75 (3)
H5	0.796 (3)	0.510 (3)	0.115 (3)	0.088*	
H6	0.939 (2)	0.529 (6)	0.049 (6)	0.088*	0.5
H6'	0.778 (5)	0.665 (3)	0.032 (6)	0.088*	0.5
O5B	0.842 (2)	0.572 (2)	0.034 (2)	0.0584 (13)	0.25 (3)

### Atomic displacement parameters (Å^2^)

**Table:**

	*U*^11^	*U*^22^	*U*^33^	*U*^12^	*U*^13^	*U*^23^
Cd1	0.02855 (11)	0.02433 (10)	0.03058 (10)	−0.00663 (6)	−0.00444 (6)	−0.00839 (6)
C1	0.0356 (9)	0.0330 (9)	0.0487 (10)	0.0015 (7)	−0.0159 (8)	−0.0100 (8)
C2	0.0369 (11)	0.0521 (12)	0.0715 (14)	−0.0020 (9)	−0.0257 (10)	−0.0205 (11)
C3	0.0335 (10)	0.0498 (12)	0.0665 (14)	−0.0143 (9)	0.0022 (9)	−0.0167 (10)
C4	0.0337 (9)	0.0371 (9)	0.0456 (10)	−0.0056 (7)	0.0011 (7)	−0.0159 (8)
C5	0.0391 (10)	0.0241 (8)	0.0487 (10)	−0.0070 (7)	−0.0070 (8)	−0.0123 (7)
C6	0.0383 (9)	0.0358 (9)	0.0452 (9)	−0.0120 (7)	−0.0064 (7)	−0.0203 (8)
N1	0.0266 (7)	0.0260 (6)	0.0375 (7)	−0.0052 (5)	−0.0057 (5)	−0.0094 (5)
N2	0.0286 (8)	0.0410 (8)	0.0470 (9)	−0.0105 (6)	−0.0073 (6)	−0.0179 (7)
C7	0.049 (5)	0.034 (4)	0.038 (4)	−0.006 (3)	−0.006 (3)	−0.002 (3)
C8	0.084 (7)	0.069 (5)	0.033 (3)	−0.022 (5)	0.004 (3)	0.005 (3)
O1	0.050 (4)	0.043 (5)	0.045 (3)	−0.017 (4)	−0.001 (3)	−0.005 (3)
O2	0.044 (5)	0.045 (4)	0.032 (3)	−0.014 (4)	−0.007 (3)	0.002 (2)
C7B	0.042 (4)	0.042 (4)	0.028 (3)	−0.005 (3)	0.009 (3)	−0.002 (3)
C8B	0.106 (10)	0.079 (8)	0.041 (4)	−0.040 (8)	0.020 (5)	−0.019 (5)
O1B	0.063 (6)	0.041 (5)	0.049 (3)	−0.018 (4)	0.013 (3)	−0.014 (3)
O2B	0.041 (4)	0.040 (3)	0.030 (3)	−0.006 (3)	−0.010 (3)	−0.002 (2)
O3	0.0278 (7)	0.0637 (10)	0.0934 (13)	−0.0077 (7)	−0.0151 (8)	−0.0249 (9)
O4	0.0829 (13)	0.0561 (10)	0.0529 (9)	−0.0055 (9)	−0.0237 (9)	−0.0168 (8)
O5	0.0560 (11)	0.0528 (12)	0.065 (3)	−0.0172 (9)	−0.0029 (15)	−0.0208 (15)
O5B	0.0560 (11)	0.0528 (12)	0.065 (3)	−0.0172 (9)	−0.0029 (15)	−0.0208 (15)

### Geometric parameters (Å, º)

**Table:**

Cd1—N2	2.2788 (15)	C6—N2	1.467 (2)
Cd1—N2^i^	2.2788 (15)	C6—H6A	0.9700
Cd1—O2B^i^	2.301 (16)	C6—H6B	0.9700
Cd1—O2B	2.301 (16)	N2—H1	0.838 (18)
Cd1—O2^i^	2.386 (16)	N2—H2	0.844 (18)
Cd1—O2	2.386 (16)	C7—O1	1.252 (9)
Cd1—N1	2.5239 (13)	C7—O2	1.276 (9)
Cd1—N1^i^	2.5239 (13)	C7—C8	1.511 (10)
C1—N1	1.482 (2)	C8—H8A	0.9600
C1—C2	1.501 (3)	C8—H8B	0.9600
C1—H1A	0.9700	C8—H8C	0.9600
C1—H1B	0.9700	C7B—O1B	1.236 (10)
C2—O3	1.417 (3)	C7B—O2B	1.275 (9)
C2—H2A	0.9700	C7B—C8B	1.522 (9)
C2—H2B	0.9700	C8B—H8B1	0.9600
C3—O3	1.421 (3)	C8B—H8B2	0.9600
C3—C4	1.512 (3)	C8B—H8B3	0.9600
C3—H3A	0.9700	O4—H3	0.834 (17)
C3—H3B	0.9700	O4—H4	0.820 (19)
C4—N1	1.475 (2)	O4—H4'	0.827 (19)
C4—H4A	0.9700	O5—H5	0.838 (16)
C4—H4B	0.9700	O5—H6	0.823 (18)
C5—N1	1.481 (2)	O5—H6'	0.827 (19)
C5—C6	1.504 (3)	O5B—H5	0.825 (17)
C5—H5A	0.9700	O5B—H6	0.838 (19)
C5—H5B	0.9700	O5B—H6'	0.842 (19)
			
N2—Cd1—N2^i^	180.0	N1—C5—H5B	109.0
N2—Cd1—O2B^i^	88.9 (4)	C6—C5—H5B	109.0
N2^i^—Cd1—O2B^i^	91.1 (4)	H5A—C5—H5B	107.8
N2—Cd1—O2B	91.1 (4)	N2—C6—C5	110.33 (14)
N2^i^—Cd1—O2B	88.9 (4)	N2—C6—H6A	109.6
O2B^i^—Cd1—O2B	180.0	C5—C6—H6A	109.6
N2—Cd1—O2^i^	86.6 (4)	N2—C6—H6B	109.6
N2^i^—Cd1—O2^i^	93.4 (4)	C5—C6—H6B	109.6
O2B^i^—Cd1—O2^i^	5.9 (8)	H6A—C6—H6B	108.1
O2B—Cd1—O2^i^	174.1 (8)	C4—N1—C5	110.11 (14)
N2—Cd1—O2	93.4 (4)	C4—N1—C1	108.91 (14)
N2^i^—Cd1—O2	86.6 (4)	C5—N1—C1	107.85 (14)
O2^i^—Cd1—O2	180.0	C4—N1—Cd1	113.82 (10)
N2—Cd1—N1	76.46 (5)	C5—N1—Cd1	100.13 (10)
N2^i^—Cd1—N1	103.54 (5)	C1—N1—Cd1	115.51 (11)
O2B^i^—Cd1—N1	89.6 (5)	C6—N2—Cd1	110.81 (11)
O2B—Cd1—N1	90.4 (5)	C6—N2—H1	107.4 (16)
O2^i^—Cd1—N1	94.4 (5)	Cd1—N2—H1	117.5 (16)
O2—Cd1—N1	85.6 (5)	C6—N2—H2	110.1 (16)
N2—Cd1—N1^i^	103.54 (5)	Cd1—N2—H2	104.7 (15)
N2^i^—Cd1—N1^i^	76.46 (5)	H1—N2—H2	106 (2)
O2^i^—Cd1—N1^i^	85.6 (5)	O1—C7—O2	124.2 (10)
O2—Cd1—N1^i^	94.4 (5)	O1—C7—C8	120.4 (9)
N1—Cd1—N1^i^	180.0	O2—C7—C8	115.3 (9)
N1—C1—C2	112.45 (16)	C7—C8—H8A	109.5
N1—C1—H1A	109.1	C7—C8—H8B	109.5
C2—C1—H1A	109.1	H8A—C8—H8B	109.5
N1—C1—H1B	109.1	C7—C8—H8C	109.5
C2—C1—H1B	109.1	H8A—C8—H8C	109.5
H1A—C1—H1B	107.8	H8B—C8—H8C	109.5
O3—C2—C1	111.52 (19)	C7—O2—Cd1	128.0 (11)
O3—C2—H2A	109.3	O1B—C7B—O2B	126.0 (10)
C1—C2—H2A	109.3	O1B—C7B—C8B	119.6 (9)
O3—C2—H2B	109.3	O2B—C7B—C8B	114.4 (9)
C1—C2—H2B	109.3	C7B—C8B—H8B1	109.5
H2A—C2—H2B	108.0	C7B—C8B—H8B2	109.5
O3—C3—C4	111.37 (18)	H8B1—C8B—H8B2	109.5
O3—C3—H3A	109.4	C7B—C8B—H8B3	109.5
C4—C3—H3A	109.4	H8B1—C8B—H8B3	109.5
O3—C3—H3B	109.4	H8B2—C8B—H8B3	109.5
C4—C3—H3B	109.4	C7B—O2B—Cd1	132.3 (10)
H3A—C3—H3B	108.0	C2—O3—C3	109.06 (16)
N1—C4—C3	110.44 (16)	H3—O4—H4	103 (3)
N1—C4—H4A	109.6	H3—O4—H4'	102 (3)
C3—C4—H4A	109.6	H4—O4—H4'	132 (7)
N1—C4—H4B	109.6	H5—O5—H6	101 (3)
C3—C4—H4B	109.6	H5—O5—H6'	101 (3)
H4A—C4—H4B	108.1	H6—O5—H6'	137 (5)
N1—C5—C6	113.08 (14)	H5—O5B—H6	101 (3)
N1—C5—H5A	109.0	H5—O5B—H6'	100 (3)
C6—C5—H5A	109.0	H6—O5B—H6'	132 (5)
			
N1—C1—C2—O3	56.0 (2)	C2—C1—N1—C5	−171.48 (16)
O3—C3—C4—N1	−59.6 (2)	C2—C1—N1—Cd1	77.53 (18)
N1—C5—C6—N2	64.5 (2)	C5—C6—N2—Cd1	−41.77 (17)
C3—C4—N1—C5	171.13 (15)	O1—C7—O2—Cd1	−18 (2)
C3—C4—N1—C1	53.06 (19)	C8—C7—O2—Cd1	163.7 (13)
C3—C4—N1—Cd1	−77.39 (17)	O1B—C7B—O2B—Cd1	−18 (2)
C6—C5—N1—C4	73.18 (18)	C8B—C7B—O2B—Cd1	160.8 (14)
C6—C5—N1—C1	−168.10 (15)	C1—C2—O3—C3	−59.3 (2)
C6—C5—N1—Cd1	−46.96 (16)	C4—C3—O3—C2	61.5 (2)
C2—C1—N1—C4	−52.0 (2)		

Symmetry code: (i) −*x*+1, −*y*, −*z*+1.

### Hydrogen-bond geometry (Å, º)

**Table:**

*D*—H···*A*	*D*—H	H···*A*	*D*···*A*	*D*—H···*A*
C4—H4*B*···O2*B*^i^	0.97	2.66	3.264 (19)	121
C5—H5*B*···O1*B*	0.97	2.63	3.425 (16)	139
N2—H1···O4^i^	0.84 (2)	2.44 (2)	3.096 (3)	136 (2)
N2—H2···O1	0.84 (2)	2.26 (2)	3.009 (16)	147 (2)
N2—H2···O1*B*	0.84 (2)	2.26 (3)	2.993 (17)	145 (2)
O4—H3···O2	0.83 (2)	1.84 (3)	2.661 (17)	167 (4)
O4—H3···O2*B*	0.83 (2)	2.02 (3)	2.837 (17)	165 (3)
O4—H4···O5^ii^	0.82 (5)	2.06 (5)	2.879 (7)	178 (7)
O4—H4···O5*B*^ii^	0.82 (5)	2.05 (5)	2.847 (18)	165 (5)
O4—H4′···O4^iii^	0.83 (2)	2.11 (3)	2.917 (4)	164 (6)
O5—H5···O1	0.84 (2)	1.99 (2)	2.828 (16)	173 (3)
O5*B*—H5···O1*B*	0.83 (2)	1.93 (2)	2.70 (2)	155 (3)
O5—H6···O5^iv^	0.82 (2)	2.09 (3)	2.895 (13)	166 (5)
O5—H6···O5*B*^iv^	0.82 (4)	1.93 (5)	2.75 (2)	173 (5)
O5—H6′···O4^ii^	0.83 (2)	2.07 (2)	2.878 (5)	168 (5)
O5*B*—H6′···O4^ii^	0.84 (2)	2.07 (2)	2.846 (17)	154 (5)

Symmetry codes: (i) −*x*+1, −*y*, −*z*+1; (ii) −*x*+1, −*y*+1, −*z*; (iii) −*x*+1, −*y*, −*z*; (iv) −*x*+2, −*y*+1, −*z*.
